# A comprehensive overview of metaplastic breast cancer: clinical features and molecular aberrations

**DOI:** 10.1186/s13058-020-01353-z

**Published:** 2020-11-04

**Authors:** Tejaswini P. Reddy, Roberto R. Rosato, Xiaoxian Li, Stacy Moulder, Helen Piwnica-Worms, Jenny C. Chang

**Affiliations:** 1 Houston Methodist Research Institute, 6670 Bertner Ave, Houston, TX 77030 USA; 2 Texas A&M Health Science Center College of Medicine, 8447 Riverside Pkwy, Bryan, TX 77807 USA; 3 Winship Cancer Institute, Emory University School of Medicine, 1365 Clifton Rd, Atlanta, GA 30322 USA; 4 The University of Texas MD Anderson Cancer Center, 1400 Holcombe Boulevard, Houston, TX 77030 USA; 5 Houston Methodist Cancer Center/Weill Cornell Medicine, OPC 24, 6445 Main Street, Houston, TX 77030 USA

**Keywords:** Metaplastic breast cancer, PI3K signaling, NOS signaling, Epithelial-to-mesenchymal transition

## Abstract

Metaplastic breast cancer (MpBC) is an exceedingly rare breast cancer variant that is therapeutically challenging and aggressive. MpBC is defined by the histological presence of at least two cellular types, typically epithelial and mesenchymal components. This variant harbors a triple-negative breast cancer (TNBC) phenotype, yet has a worse prognosis and decreased survival compared to TNBC. There are currently no standardized treatment guidelines specifically for MpBC. However, prior studies have found that MpBC typically has molecular alterations in epithelial-to-mesenchymal transition, amplification of epidermal growth factor receptor, PI3K/Akt signaling, nitric oxide signaling, Wnt/β-catenin signaling, altered immune response, and cell cycle dysregulation. Some of these molecular alterations have been studied as therapeutic targets, in both the preclinical and clinical setting. This current review discusses the histological organization and cellular origins of MpBC, molecular alterations, the role of radiation therapy, and current clinical trials for MpBC.

## Introduction

Metaplastic breast cancer (MpBC) is a rare and aggressive malignancy that accounts for 0.2–5% of all breast cancers, and as such, MpBC carries the worst prognosis in comparison to other breast cancer types and plays a significant role in global breast cancer mortality [1]. In the SEER database from 1973 to 2015, there were less than 10,000 cases of MpBC in the USA annually [2]. This malignancy is characterized by the histological presence of at least two cellular types, typically epithelial and mesenchymal components [3]. MpBC is typically a triple-negative breast cancer (TNBC), meaning the tumor lacks the expression of estrogen receptor (ER), progesterone receptor (PR), and human epidermal growth factor 2 receptor (HER2) [3]. Unfortunately, MpBC carries a worse prognosis in comparison to non-metaplastic TNBC, has twice the risk of recurrence, and has a shorter disease-free and overall survival (OS) [4]. The term “metaplastic carcinoma” was first published by Huvos and colleagues in 1973 [5]. Due to its rare and aggressive nature, there have been many limitations in completely delineating the molecular and genetic landscape of MpBC. However, an emerging number of MpBC case studies, enhanced technology in research, and increased awareness of this rare breast neoplasm have allowed clinicians and researchers to gain a better understanding of the morphology, prognosis, molecular alterations, and potential treatment options. The goal of this review is to provide an overview of MpBC, including cellular and molecular characteristics, histopathology, treatment options, and ongoing clinical trials.

## Histological organization of MpBC

By definition, metaplastic carcinomas contain one or more cell populations that have undergone metaplastic differentiation, meaning that cells have converted from glandular to non-glandular morphology [6]. These metaplastic changes include carcinomatous (squamous) and sarcomatous elements, including osseous, chondroid, and spindle morphology [1]. The WHO Classification of Breast Tumors classifies MpBC as mixed metaplastic carcinoma, low-grade adenosquamous carcinoma, fibromatosis-like, squamous cell carcinoma, spindle cell carcinoma, and metaplastic carcinoma with mesenchymal differentiation [1, 7]. All of these metaplastic variants are aggressive and chemoresistant and have a high propensity to metastasize, except fibromatosis-like carcinoma and low-grade adenosquamous carcinoma [8].

Understanding the differences between histological variants may provide insight into clinical prognosis and potential therapeutic options. For example, Schwartz and colleagues organized MpBC into epithelial and mixed types, in which epithelial types included squamous cell carcinoma, adenocarcinoma with spindle cell differentiation, and adenosquamous carcinoma, whereas mixed types included carcinoma with chondroid metaplasia, carcinoma with osseous metaplasia, and carcinosarcoma [9]. Tse and colleagues classified MpBC into three groups, epithelial-only carcinoma, biphasic epithelial and sarcomatoid carcinoma, and monophasic spindle cell carcinoma [10]. Oberman conducted a clinicopathological study of 29 patients with primary breast neoplasms and classified MpBC into spindle cell carcinoma, invasive ductal carcinoma with extensive squamous metaplasia, and invasive ductal carcinoma with pseudosarcomatous metaplasia [11]. One of the primary issues with these descriptive classifications is the lack of correlation between the microscopic pattern and prognosis of the disease, in part due to the rarity of the disease.

Of these, one publication by Song et al. found that there was clinical significance to sub-classifying MpBC [12]. This study showed that the prognosis of MpBC was worse than triple-negative invasive ductal carcinoma (TN-IDC), with a 5-year overall survival rate of 54.5% vs. 73.3%, respectively. Adenocarcinoma with spindle cell differentiation had the worst 5-year overall survival rate at 40%. Overall, this study concluded that separating MpBC based on histological variants may have clinical significance.

The prognosis and response to therapy vary among the metaplastic variants. MpBC also shows increased locoregional and distant tumor recurrence and is far more aggressive than invasive ductal carcinoma, even when matched for age, stage, and tumor grade [13]. In addition, the majority of MpBC is triple-negative, yet the genomic, transcriptomic, and proteomic characteristics between MpBC and TNBC differ. Examples of this include the marker p63, which is commonly expressed in spindle cell carcinoma, or that low-grade adenosquamous carcinoma harbor high rates of *PIK3CA* mutations, but no *TP53* mutations and androgen receptor expression. Conventional TNBC has a low rate of *PIK3CA* mutations [14, 15].

A genomics profiling study performed by McQuerry and colleagues found that MpBC samples with mesenchymal (osteoid or chondroid) histology had increased Snail, BCL-2-like-1 protein, and Akt1 pathway activity in comparison to non-mesenchymal MpBC tumors [16]. When comparing the gene expression profiles of MpBC to TNBC tumors, MpBC tumors had more upregulation of epithelial-to-mesenchymal transition (EMT) and collagen genes, but downregulation of late cornified envelope and keratinization genes. Overall, these results support that MpBC histological variants may exhibit different genomic profiles and that EMT may play an influential role in the aggressiveness and lethality of this rare breast cancer subset.

A proteomics study by Djomehri and colleagues discovered potential metaplastic pathological subtype-specific biomarkers/therapeutic targets as well as proteomic differences between MpBC and TNBC [17]. In this study, they performed multiplex quantitative tandem mass tag-based proteomics and gene set enrichment analysis to elucidate unique protein signatures in TNBC, MpBC pathological variants (spindle, squamous, and sarcomatoid MpBC), and normal mammary tissue. Compared to the TNBC proteome, the top upregulated pathway in the MpBC proteome was EMT and the top downregulated pathway was oxidative phosphorylation (OXPHOS). When proteomes of the specific MpBC pathological subtypes were compared, they discovered that spindle MpBC was highly enriched with MYC and E2F targets and ribosomal pathway proteins. Squamous MpBC had elevated interferon-gamma signaling/broad inflammatory responses, TP53 and PI3K signaling, apical junction signaling, and decreased OXPHOS, MYC, and E2F targets. Sarcomatoid MpBC displayed high EMT and OXPHOS signaling and low PI3K/MTORC1 and interferon-gamma signaling. The discovery of unique proteomic features among the MpBC pathological variants may provide new insight into novel treatment strategies to improve the survival of MpBC patients. Nevertheless, more studies and discussions are needed to examine the clinical value of separating MpBC based on the histological variant.

## Cell origin of MpBC

The clonality and origin of MpBC have been debated for years, and there are at least three hypotheses to explain why MpBC are biphasic tumors, meaning the presence of sarcomatous and carcinomatous components within the same tumor. The collision theory suggests that carcinomatous and sarcomatous tissues are derived from separate progenitor cells [9], while the combination theory of monoclonal origin suggests that a common multipotent progenitor cell is responsible for giving rise to both sarcomatous and carcinomatous cells. The conversion/metaplastic theory suggests that the sarcomatous components derive from the carcinomatous component via a metaplastic process. Evidence to support the metaplastic theory comes from data that showed both epithelial and mesenchymal components of the tumor display a positive expression of cytokeratin, S-100, and vimentin [18]. Furthermore, it has also been suggested that MpBC may derive from myoepithelial cells, as the tumors are frequently positive for myoepithelial markers including CD10, p63, and smooth muscle actin [19].

## Molecular alterations in MpBC

Despite its rarity, there have been some studies that have revealed molecular alterations and actionable genetic changes within MpBC. Molecular analysis of MpBC will be crucial to identify potential options for targeted therapeutic intervention and for promising clinical strategies. Here, we provide an overview of molecular alterations and characteristics found in MpBC cases, which is also summarized in Table 1.

**Table 1:** List of molecular alterations in MpBC

Molecular alteration	Description	Reference	Samplesize
Epithelial-to-mesenchymal transition (EMT)	-EMT core genetic signature shares similarity to core geneticsignatures of claudin-low and metaplastic breast cancers [20]. -MpBC tumors have more stem-like features, express highlevels of EMT markers, and share a similar genetic signature totumor-initiating cell (TIC) genetic signature [21]. -TICs are prominent following endocrine/chemotherapy, morechemoresistant, exhibit EMT, and can undergo self-renewal [22].	Taube et al. [20]	244
		Hennessy et al. [21]	28
Epidermal growth factor receptor(EGFR) signaling pathway	-34% of MpBC cases exhibit EGFR gene amplification associatedwith gene overexpression and no EGFR activating mutations [23, 24]. -Fluorescent in situ hybridization showed high EGFR copynumber secondary to aneusomy (22%) and amplification (4%) [25]. -Majority of MpBC is positive for p63 (59%), cytokeratin 5/6 (58%),KIT (24%), and EGFR (66%) overexpression [25].	Reis-Filho et al. [23]	25
		Reis-Filho et al. [24]	47
		Gilbert et al. [25]	38
Phosphoinositide 3-kinase (PI3K)signaling pathway	-47% of MpBC tumors harbor *PIK3CA* mutations and 5% have *PTEN*deletions [21]. 36.4% of HR+ breast cancers have *PIK3CA* mutations [26]. -Whole-exome sequencing analysis of MpBC tumors showedthe most altered genes were *PIK3CA* (29%), *PIK3R1* (11%), *FAT1*(11%), *ARID1A* (11%), and *PTEN* (11%) [27]. -Next-generation sequencing of MpBC tumors showed themost commonly altered genes were *TP53* (68.4%), *PIK3CA*(42.1%), and *PTEN* (15.8%) [28].	Hennessy et al. [21]	28
		Razavi et al. [26]	1918
		Ng et al. [27]	35
		Afkhami et al. [28]	21
Nitric oxide synthase (NOS)signaling pathway	-TNBC expresses high levels of nitric oxide (NO) than HER2+or luminal breast cancers and enhanced inducible nitric oxidesynthase (iNOS) expression is associated with worse prognosisand may confer resistance to chemotherapy [29]. -Inhibition of iNOS via L-NMMA in combination with docetaxelis more effective than docetaxel alone in enhancing tumorapoptosis, cell proliferation/migration, and reducing tumor-initiating capacity in TNBC and MpBC models. -39/40 (97.5%) of MpBC tumors harbor a *RPL39 A14V* oncogenicmutation, which is associated with enhanced NO activity, cancercell stemness, and lung metastasis [30].	Granados-Principalet al. [29]	83
		Dave et al. [30]	40
Wnt/β-catenin signaling	-Immunohistochemistry (IHC) of MpBC samples showed aberrant β-catenin expression in 33/36 (92% of cases), and mutational analysisshowed that 25.9% of MpBC tumors had *CTNNB1* missense mutations,7.4% tumors had *APC* mutations, and 18.5% tumors had *WISP3*mutations [31]. -IHC of MpBC tumor samples reveals that β-catenin expressionhas more focal nuclear localization [32]. -MpBC tumors commonly harbor mutations in Wnt/β-cateninsignaling and PI3K/Akt signaling than TNBC tumors [27]. -The levels of CCN6 are low in MpBC, leading to enhancedinsulin-like growth factor 1 levels, EMT, invasion, metastasis, andbone morphogenic-4 signaling [8]. -A mouse model of mammary epithelium-specific Ccn6 proteindeletion (MMTV-cre;Ccn6^fl/fl^) has been developed, which canrecapitulate many features of human spindle MpBC tumors [17, 33].	Hayes et al. [31]	26
		Lacroix-Triki et al. [32]	52
		Ng et al. [27]	35
		Martin et al. [33]	–
Programmed cell death protein 1 (PD-1)/programmed death ligand-1 (PD-L1)	-PD-L1 is expressed more in MpBC tumors (46%) relative to otherbreast tumor types (6% in HR+ and 9% in HER2+ breast cancers [34]. -Another study performed PD-L1 immunohistochemical staining of 21MpBC tumor samples and found that PD-L1 expression was associatedwith a worse RFS and OS [28]. -A patient with metastatic MpBC (PD-L1+ and with *PIK3CA*H1047L mutation) showed a dramatic response topembrolizumab in combination with nab-paclitaxel [35].	Joneja et al. [34]	290
		Afkhami et al. [28]	21
		Adams 2017 [35]	1
Cell cycle regulation	-MpBC tumors harbor a high frequency of *TP53* (64%) and *TERT*(catalytic subunit of telomerase) promoter mutations (25%) [36] -*TERT* mutations are commonly found in the spindle andsquamous MpBC [36] -Myoepithelial MpBC shows a 9p21.3 chromosomal loss, including lossof genes CDK2NA and CDK2NB, which code for cyclin-dependent kinaseinhibitors p16^INK4a^ and p15^INK4b^ [37]. -64.3% of myoepithelial MpBC tumors with 9p21.3 loss alsohad a *PIK3CA* mutation [37].	Krings and Chen [36]	28
		Bartels et al. [37]	34

### Epithelial-to-mesenchymal transition

Epithelial-to-mesenchymal transition (EMT) is a transient process in which epithelial cells lose their cell polarity and cell-cell adhesion qualities and acquire mesenchymal properties, including enhanced migratory capacity, resistance to apoptosis and chemotherapy, invasiveness, and characteristic morphological and gene expression changes [38]. EMT after embryogenesis is considered pathological, and this process is associated with a loss of E-cadherin and claudin expression and enhanced expression of mesenchymal markers such as vimentin and smooth muscle cell actin [39]. EMT is typically regulated by transcription factors (TF) such as Goosecoid, Snail, Slug, Twist, FOXC1, FOXC2, Zeb1, and Zeb2 [20]. While EMT has been extensively studied in claudin-low and metaplastic breast cancers, EMT has also been involved in tumorigenesis of other cancers, such as liver, lung, prostate, pancreas, thyroid, and glioblastoma multiforme [40, 41]. In general, tumors that undergo EMT typically harbor an epithelial phenotype and have activated EMT-TF-dependent cellular processes, such as dedifferentiation and plasticity [41]. Taube and colleagues conducted a study in which they identified an EMT core genetic signature that was enriched for genes regulated by Zeb1 and this genetic signature was similar to that of claudin-low and metaplastic breast cancers [20]. Hennessy et al. found comparable results in their study, in which 28 MpBC tumor samples were transcriptionally profiled and probed using a tumor-initiating cell (TIC) gene signature [21]. This TIC gene signature was developed by Creighton et al. [22]. MpBC tumors shared a similar genetic signature to TIC and claudin-low breast cancer gene signatures, had more stem-cell-like features, and expressed high levels of EMT markers [21]. TICs are more prominently observed following endocrine therapy or chemotherapy, have intrinsic resistance to chemotherapy, exhibit EMT characteristics, and are capable of self-renewal [22]. A crucial finding from this study was that MpBC showed a high frequency of amplification, mutation, and activation of phosphoinositide 3-kinase (PI3K) signaling relative to basal and claudin-low breast cancers. Therefore, EMT/stem-cell-like features in combination with PI3K signaling hyperactivation may provide an explanation for why MpBC is an aggressive, chemorefractory subtype and potentially originated from a chemoresistant stem cell.

### Epidermal growth factor receptor (EGFR)

**Fig. 1:**
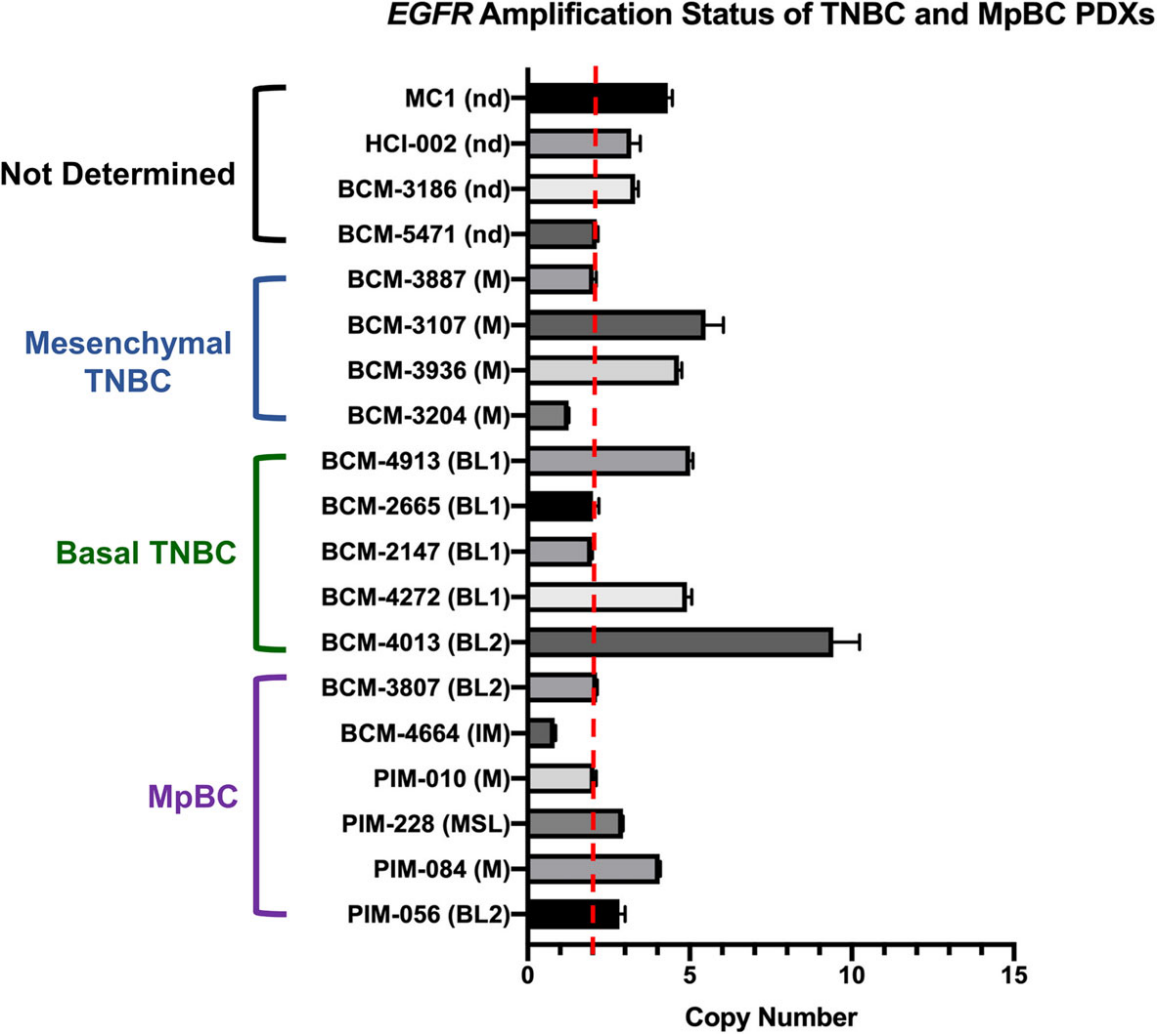
EGFR copy number variant values of mesenchymal TNBC, basal TNBC, and MpBC PDX tumors. Droplet digital PCR was performed using DNA isolated from PDX tumors, and *EGFR* and *RPP30* (reference gene)-specific primers and probes. Red dotted line indicates the normal copy number threshold (CN 2). Pietenpol Classification [42]: BL1, basal-like 1; BL2, basal-like 2; IM, immunomodulatory; LAR, luminal androgen receptor; M, mesenchymal; MSL, mesenchymal-stem like; nd, not determined

MpBC consistently overexpresses EGFR, but usually lacks HER2 overexpression and amplification [23]. Reis-Filho and colleagues found that 34% of MpBC cases exhibit EGFR gene amplification and this gene amplification is associated with gene overexpression [24]. This study found no activating mutations in EGFR, suggesting that point mutations within the receptor are unlikely to influence the overexpression of EGFR. Another study assessed 77 MpBC samples and found that the majority of the samples were positive for p63 (59%), cytokeratin 5/6 (58%), EGFR overexpression (66%), and KIT (24%) [25]. This study also found no activating mutations in EGFR and KIT. Fluorescence in situ hybridization was performed to show high *EGFR* copy number secondary to aneusomy (22%) and amplification (4%). To compare the differences in *EGFR* amplification among MpBC and mesenchymal and basal TNBC tumors, we performed droplet digital PCR analysis (ddPCR) to assess *EGFR* DNA copy number values. We used DNA isolated from well-established MpBC and TNBC patient-derived xenograft (PDX) tumors. The details of the experiment are included in the supplementary information. We found that in comparison to mesenchymal TNBC and MpBC, basal TNBC had the highest *EGFR* copy number values (Fig. 1). PDX BCM-4013 (basal-like 2 subtype, BL2) exhibited the most *EGFR* amplification among all PDXs. BL2 TNBC tumors have been shown to exhibit enhanced EGFR gene expression [42]. Fifty percent of the MpBC PDX tumors exhibited *EGFR* copy number values greater than two. These findings shed light on the differential expression of *EGFR* between TNBC and MpBC as well as warrant further investigation on using EGFR tyrosine kinase inhibitors as therapeutics against MpBC.

### Phosphoinositide 3-kinase (PI3K) pathway

**Fig. 2:**
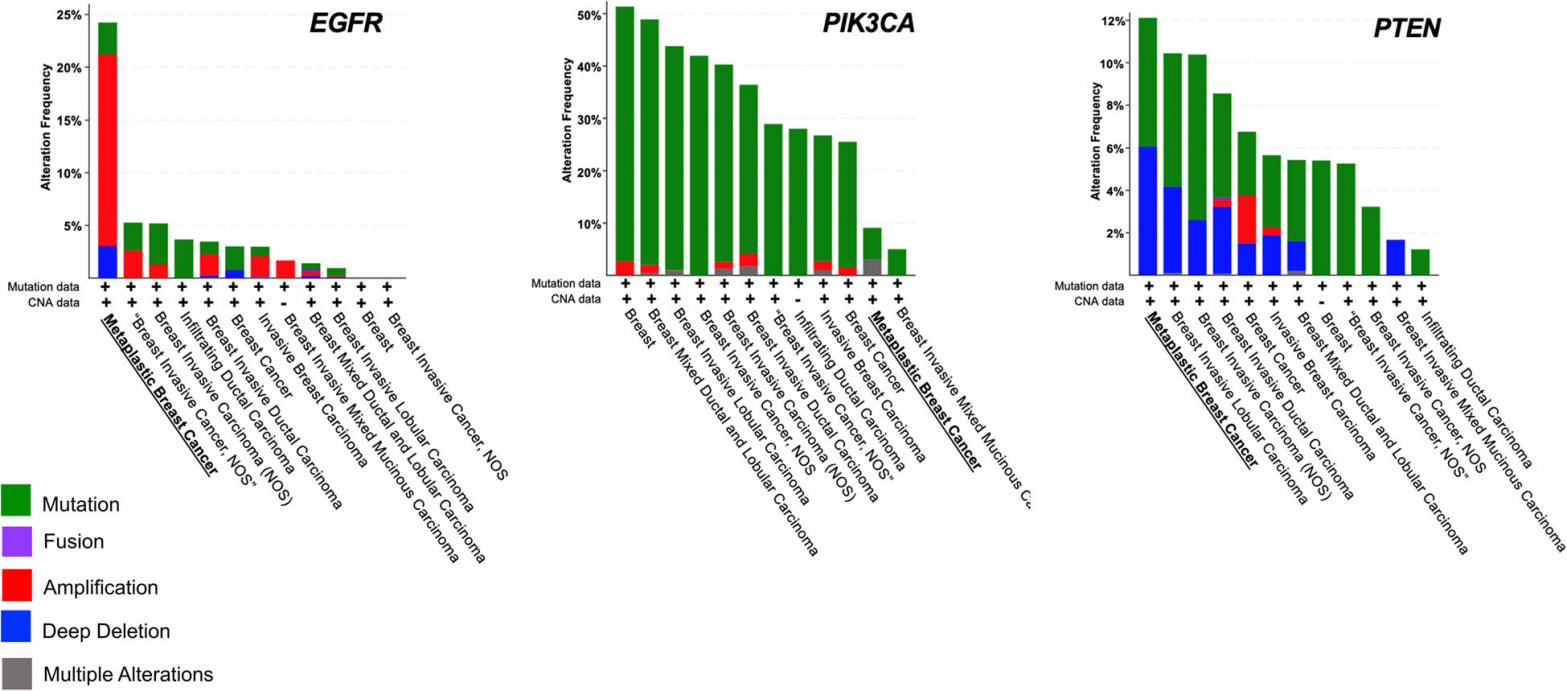
MpBC harbors genetic alterations in *EGFR*, *PIK3CA*, and *PTEN* genes. Data derived from cBioPortal database of 9052 patients across 12 breast cancer studies

**Fig. 3:**
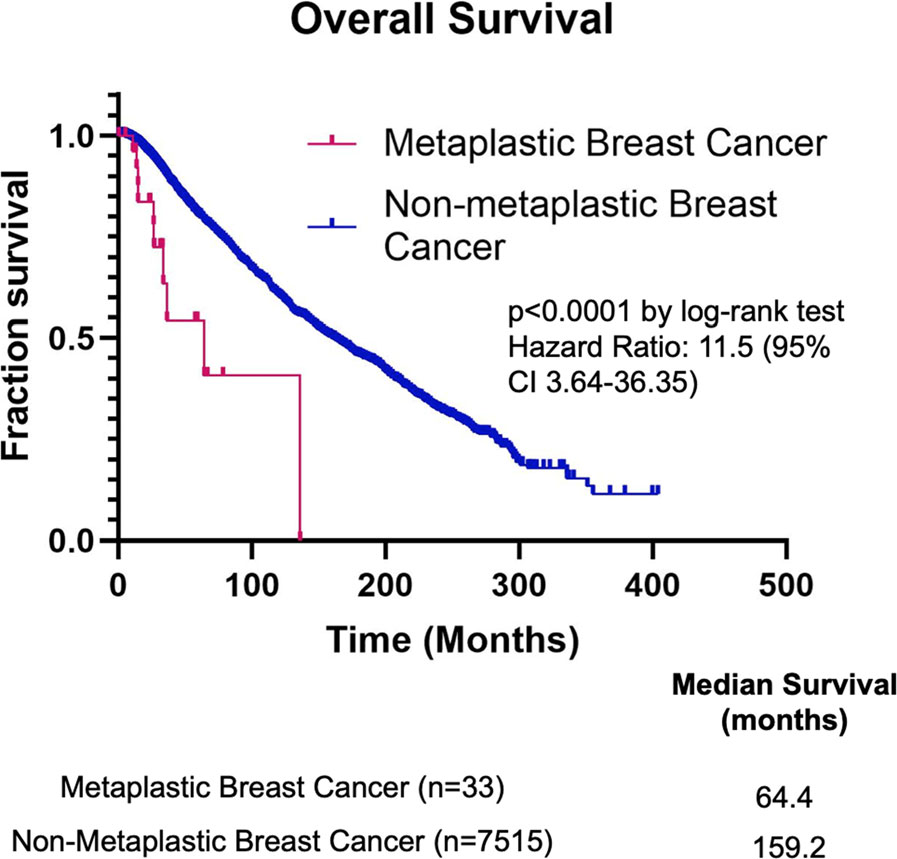
Overall survival curve for patients with metaplastic and non-metaplastic breast cancers. Data derived from cBioPortal database of 7548 patients across 12 breast cancer studies

The PI3K/Akt/mTOR signaling pathway controls cellular growth, proliferation, metabolism, cellular survival, and angiogenesis and is one of the most frequently dysregulated pathways in cancer [43]. The pathway can be hyperactivated in cells through diverse genomic alterations, such as mutations in *PIK3CA*, *PIK3R1*, *AKT1*, *MTOR*, *TSC2*, and *LKB1*, as well as various other tumor suppressor genes and oncogenes [44]. *PIK3CA* codes for p100α, the catalytic subunit of the PI3Kα complex, and phosphatase and tensin homolog (*PTEN*) is a tumor suppressor gene of this pathway that is frequently deleted in cancers. A seminal study in the field of MpBC research found that this rare cancer type harbors ~ 47% *PIK3CA* mutations and ~ 5% *PTEN* deletions [21]. MpBC tumors harbor more *PIK3CA* mutations than hormone receptor (HR)+ breast cancers, in which 36.4% of those cancers have a *PIK3CA* mutation [26]. However, the increased percentage of MpBC with *PIK3CA* mutations may be a biased result, as this study had a small sample size of MpBC tumors. Nevertheless, another study conducted whole-exome sequencing on MpBC samples and found that MpBC tissue contained genetic alterations in *PIK3CA* (29%), *PIK3R1* (11%), *FAT1* (11%), *ARID1A* (11%), and *PTEN* (11%) [27]. A next-generation sequencing mutational assay on tumor samples from 19 MpBC patients found comparable results and showed that the most commonly altered genes in MpBC were *TP53* (68.4%, 13/19), *PIK3CA* (42.1%, 8/19), and *PTEN* (15.8%, 3/19) [28]. The study also showed that recurrence-free survival (RFS) and OS were significantly worse for MpBC patients with *PIK3CA* mutations. We analyzed a publicly available cancer cohort (*n* = 9052) from cBioPortal to identify alteration frequencies for *EGFR*, *PIK3CA*, and *PTEN* genes across all breast cancer subtypes (Fig. 2). Across all cancer types, MpBC showed the highest frequency of alterations in *EGFR* and *PTEN*, and a modest percentage of *PIK3CA* mutations. While it is difficult to conclude whether *PIK3CA* mutations are more commonly seen in MpBC vs. TNBC, largely due to the rare nature of MpBC, we suggest that targeting this pathway with isoform-specific inhibitors in combination with other novel therapeutics may be the future for MpBC treatment. From the same database, we also compared the OS of 33 MpBC patients to 7515 non-metaplastic breast cancer patients (including HR+, HER2+, and TNBC) and found MpBC had a poorer OS (HR 11.5, 95% CI 3.64–36.35) than non-metaplastic breast cancer (Fig. 3). The median survival rate for MpBC patients was 64.4 months, versus 159.2 months for patients diagnosed with non-metaplastic breast cancers. These findings further support studies that have described MpBC as highly aggressive cancer with poorer clinical outcomes than other breast malignancies [4, 45, 46].

### Nitric oxide signaling pathway

TNBC expresses higher levels of nitric oxide (NO), which is estimated by nitrate levels, than HER2+ or luminal breast cancers [47]. Furthermore, previous in vitro studies have shown that TNBC tumors expressing inducible nitric oxide synthase (iNOS) produce moderate to high levels of NO, and the increased iNOS activity may confer resistance to chemotherapy [48]. A preclinical study demonstrated that combining docetaxel with pan-NOS inhibitor NG-monomethyl-l-arginine acetate (L-NMMA) in a xenograft model of MDA-MB-231 decreased Ki67 proliferating cells, enhanced tumor apoptosis, and reduced tumor-initiating capacity of residual tumor cells after chemotherapy to a larger degree than docetaxel alone [29]. Another preclinical study assessed the potential of L-NMMA to sensitize metaplastic breast cancer to docetaxel [30]. In this study, L-NMMA significantly reduced cell migration and proliferation in a dose-dependent manner in MpBC cell lines, Hs578T, and BT549. L-NMMA also significantly enhanced docetaxel-mediated apoptosis in MpBC cell lines, as evidenced by a greater number of Annexin V-positive cells found in cell lines treated with L-NMMA + docetaxel combination therapy, versus vehicle control or monotherapy. This study also examined tumor samples from patients with histologically confirmed MpBC and found that out of 40 MpBC samples, 39 samples had a *RPL39 A14V* mutation. The *RPL39 A14V* mutation is associated with enhanced NO activity and cancer cell stemness. MpBC tumors that harbored a RPL39 mutation and were resistant to docetaxel became sensitized to the taxane by L-NMMA through inhibition of STAT3 signaling. Taking this into consideration, a phase 1b/2 clinical trial is being conducted to assess the maximum-tolerated dose, dose-limited toxicities, recommended phase 2 dose, and efficacy of L-NMMA in combination with docetaxel for refractory locally advanced and metastatic triple-negative breast cancer patients (NCT02834403). This clinical trial is also recruiting patients diagnosed with MpBC.

### Wnt/β-catenin pathway

The Wnt/β-catenin pathway plays a vital role in embryonic development, EMT, and carcinogenesis [32]. In a 2008 study, 36 MpBC samples were analyzed for alterations in the Wnt signaling pathway, by examining immunohistochemical (IHC) stains and mutation analysis of key proteins in the pathway [31]. IHC showed aberrant β-catenin expression in 33/36 cases (92%). Mutational analysis demonstrated that 25.9% of samples had *CTNNB1* missense mutations, in the region coding for the NH2-terminal domain of β-catenin, likely impairing its ability to undergo degradation. *APC* and *WISP3* mutations were seen in 7.4% and 18.5% of samples respectively. A subsequent study found a lack of *CTNNB1* mutations in MpBC samples, but more enhanced and focal nuclear localization of β-catenin in MpBC samples via IHC [32]. A more recent study conducted whole-exome sequencing of 35 MpBC samples and compared the MpBC genomic landscape to that of TNBC from The Cancer Genome Atlas [27]. The study found that MpBC more commonly harbored mutations in the Wnt signaling and PI3K/Akt/mTOR pathway than TNBC. These mutations rendered both pathways to be more hyperactive in MpBC. These correlative studies that suggest the Wnt pathway plays a role in MpBC disease pathogenesis have been translated into preclinical studies, particularly with the development of a mouse model of mammary epithelium-specific Ccn6 protein deletion (MMTV-cre;Ccn6^fl/fl^) [33]. *CCN6*, also known as *WISP3*, is a matricellular protein involved in development during chondrogenesis, skeletogenesis, and cell attachment to the extracellular matrix. In aggressive cancers such as MpBC, levels of CCN6 are low, which leads to enhanced EMT, invasion, metastasis, insulin-growth factor-1, and bone morphogenic-4 signaling [8]. Studies using the MMTV-cre;Ccn6^fl/fl^ model have yielded promising results to suggest that Wnt and IGF-signaling may work synergistically to regulate MpBC tumorigenesis.

### Programmed cell death protein 1 (PD-1)/programmed death ligand-1 (PD-L1)

A study examined 290 tumor tissues of HR+ and HER2+ breast cancers, TNBC, and MpBC for immunohistochemical staining of PD-1 on tumor-infiltrating lymphocytes (TILs) and PD-L1 in breast cancer cells [34]. The study found a substantially enhanced expression of PD-L1 on MpBC tumor tissues (46%) relative to all other tumor tissues (6% in HR+ and HER2+, 9% in TNBC). There was enhanced variability in the expression of PD-1 on TILs in MpBC. Coinciding with this study, a case report published on a patient with metastatic MpBC, from an ongoing clinical trial (NCT02752685), showed a dramatic response to pembrolizumab (anti-PD1) in combination with nab-paclitaxel [35]. PD-L1 staining of the tumor biopsy at baseline showed 100% of tumor cells were positive for PD-L1, and another staining showed increased TIL infiltration after pembrolizumab treatment. The case report described how the PI3K/Akt and Ras-MAPK pathways play a role in regulating immune evasion and that the patient’s tumor harbored a hyperactivating *PIK3CA* mutation (H1047R), which may have influenced tumoral PD-L1 expression. Furthermore, EMT, which is a common phenomenon in MpBC, may also influence PD-L1 expression. Another study performed PD-L1 immunohistochemical staining of 21 MpBC tumor samples and found that PD-L1 expression was associated with a worse RFS and OS (HR 1.08, 95% CI 1.01–1.16 and 1.05, 95% CI 1.00–1.11, respectively) [28]. Overall, these findings describe the strong potential of immune checkpoint inhibitors in the treatment arsenal for MpBC. Some of the clinical trials described in Table 2 are taking advantage of immunotherapy as a promising therapeutic for MpBC (NCT02834013, NCT02752685).

### Cell cycle regulating proteins

Krings and Chen conducted a study in which they sequenced 408 cancer-related genes in 28 MpBC samples, and they found that MpBC harbored a high frequency of *TP53* (64%) and *TERT* (catalytic subunit of telomerase) promoter mutations (25%), but the latter frequency varied among MpBC subtypes [36]. *TERT* promoter mutations were enriched in the spindle cell and squamous cell carcinoma variants (47%). Furthermore, the percentage of *TP53* mutations in this study was comparable to another study, which found MpBC to harbor 69% *TP53* mutations [27]. A study examined myoepithelial MpBC and found that most cases (28 out of 34, 82.4%) showed a distinct chromosomal loss in 9p21.3, including loss of *CDK2NA* and *CDK2NB* [37]. *CDKN2A* and *CDKN2B* code for the proteins p16^INK4a^ and p15^INK4b^, respectively, which function as inhibitors of Cdk4 and Cdk6 and can induce G1 cell cycle arrest by inhibiting the phosphorylation of retinoblastoma protein [49]. Another finding was that loss of 9p21.3 in 64.3% of all MpBC tumor samples was accompanied by a concurrent *PIK3CA* mutation.

## The role of systemic therapy

MpBC is likely to present with more locally advanced disease and a poorer prognosis in comparison to TNBC [50]. The current standard of care for MpBC follows the same guidelines as TNBC, yet MpBC responds poorly to most systemic chemotherapy and has poorer clinical outcomes than TNBC [50–52]. Chen et al. examined 46 MpBC cases and found that the partial response rates for MpBC patients receiving neoadjuvant chemotherapy and first-line chemotherapy were 18.2% and 8.3%, respectively [50]. None of the patients responded to anthracycline, cyclophosphamide, and vinorelbine-based therapies, but a small cohort of the MpBC patients exhibited a partial response to taxane-based therapy. Aydiner and colleagues conducted an observational study assessing the survival and response to treatment of 54 MpBC and 51 TNBC patients [46]. The study found that MpBC patients had a decreased response to neoadjuvant chemotherapy (anthracycline and taxane-based therapy) than TNBC patients (12.5% vs. 75%) and that none of the MpBC patients achieved a complete response to neoadjuvant chemotherapy. In comparison to TNBC, MpBC tends to present with a larger primary tumor size, less nodal involvement, higher histological grade, and heterogeneity, as well as p53 and Ki-67 overexpression [52]. These characteristics may contribute to why MpBC is more chemorefractory than TNBC.

Despite many studies suggesting that MpBC is more chemorefractory than TNBC, there is a subset of MpBC patients that may benefit from systemic therapy. Schroeder and colleagues conducted a retrospective study of a MpBC study cohort from the Surveillance, Epidemiology, and End Results Program (SEER) database [53]. The study cohort included 1516 patients diagnosed with MpBC and 220,375 patients with invasive ductal carcinoma (IDC). Of the MpBC cases examined, 64.1% were TNBC, 5.2% were HER2+, and 23.0% were HER2−/HR+ breast cancers. The 3-year OS for HER2+ MpBC was significantly greater than for triple-negative or HR+ MpBC (91.8% vs. 75.4% vs. 77.1%, *p =* 0.025). Using a multivariate Cox proportional hazards model, the study showed that there was no statistical difference between the OS for HER2+ MpBC cases and HER2+ IDC cases (hazard ratio = 1.16, 95% CI 0.48–2.81, *p* = .734). Although HER2+ MpBC cases were only a small portion of cases in this study (5.2%), these findings suggest that systemic therapy, particularly HER2 targeted therapies, may provide improved survival benefit for HER2+ MpBC. Furthermore, this study also suggests that a broader approach to therapeutic options is warranted with MpBC and that systemic therapies may be efficacious for a particular subset of MpBC patients.

## Radiation therapy

There have been limited studies and guidelines regarding the use of radiation therapy (RT) in the adjuvant setting for MpBC, and unfortunately, the published studies have patient cohorts that are generally small. Tseng and Martinez conducted a retrospective study in which they investigated a cohort of MpBC patients treated from 1998 to 2006 (SEER database) and found that RT improved the OS of MpBC patients following lumpectomy or mastectomy [54]. A case series studying 18 patients with MpBC showed that patients who underwent postoperative RT had longer overall survival than patients who did not receive RT [55]. A retrospective cohort study by Li and colleagues in 2019 investigated 2267 patients diagnosed with MpBC between 1998 and 2015 from the SEER database and found that MpBC patients who received RT had a better OS and breast cancer-specific survival compared to those not treated with RT, and this effect was seen particularly in large tumors and elderly patients [56]. However, these studies must be analyzed with caution as either these are retrospective studies, or the studies have a small sample size. To obtain a deeper understanding of how RT can truly benefit MpBC patients in the adjuvant setting, it would be valuable to conduct prospective studies with sufficient sample sizes and develop standardized RT guidelines.

## Potential therapies in clinical trials

Despite the current treatment status for MpBC, which follows a one-size-fits-all scheme in which MpBC patients are provided the same therapeutic options to TNBC patients, there have been completed and ongoing clinical trials specifically on targeted therapeutics against MpBC. For example, a phase 1 clinical trial was conducted to assess the safety and efficacy of mTOR inhibition with temsirolimus or everolimus in combination with VEGF inhibitor bevacizumab and liposomal doxorubicin in 52 patients with advanced MpBC [57]. The objective response rate (ORR) was 21% (complete response = 4 [8%]; partial response = 7 [13%], and 10 patients [19%] had stable disease for at least 6 months, with a clinical benefit rate (CBR) of 40%). Forty-two tumor samples were available for genetic testing and 32 (74%) had a *PIK3CA* mutation. When the presence of a *PIK3CA* mutation was taken into consideration, they found a significant improvement in ORR (31% vs. 0%), but no improvement in CBR (44% vs. 45%, *p* > .99). Though a limitation of this study is the sample size, largely due to the rare nature of MpBC, another point to note is that the *PIK3CA* mutational analysis was specific to hotspot mutations. There is a chance that the tumor samples of the patient cohort may also harbor other PI3K/Akt pathway alterations that could not be detected via molecular testing. A case report was published in 2019 about a patient diagnosed with MpBC (H1047R *PIK3CA* mutation +), who enrolled in the BELLE-4 clinical trial, and achieved a durable response and overall survival of 42 months to combination therapy of paclitaxel and buparlisib, a pan-PI3K inhibitor [58]. In addition, immune checkpoint blockade, specifically a combination of nivolumab (anti-PD1) and ipilimumab (anti-CTLA4), has shown efficacy in a small cohort of MpBC patients (The DART trial) [59]. In this prospective, single-arm, phase 2 trial, 17 MpBC patients received combination therapy. The ORR% in the study was 12% (RECIST 1.1) and 18% (iRECIST), with three patients showing ongoing responses at 27, 25, and 23 months, respectively. These smaller studies are first to shed light on the potential of targeting the PI3K pathway and utilizing immunotherapy as treatment options for MpBC. Table 2 provides a list of ongoing clinical trials recruiting patients diagnosed with MpBC.

**Table 2:** List of ongoing clinical trials recruiting patients with MpBC

Trial	Phase	Status
ARQule: ARQ751 (pan-AKT inhibitor) plus fulvestrant or paclitaxel compared to ARQ751 plus placebo in patients with breast or endometrial cancer harboring *PIK3CA*/AKT/PTEN mutations (NCT02761694)	1b	Ongoing
ARTEMIS: A clinical trial implementing diagnostic imaging + tumor genetic signature to predict sensitivity to standard-of-care versus personalized therapy. A non-randomized trial in which patients undergo baseline imaging and molecular testing of tumor biopsy. They receive standard anthracycline-based chemotherapy and undergo ultrasound imaging after cycles 2 and 4. After completing cycle 4 and obtaining molecular testing results, the patient may elect to continue standard chemotherapy or proceed to an experimental clinical trial designed to match tumor profile and TNBC subtype. Patients with tumors predicted to be resistant to standard chemotherapy are advised to participate in the experimental clinical trial (NCT02276443)	N/A	Ongoing
L-NMMA (pan-nitric oxide synthase) inhibitor plus docetaxel in refractory locally advanced or metastatic TNBC patients (NCT02834403)	1b/2	Ongoing
DART - Dual anti-CTLA4 and anti-PD1 blockade in rare tumors: Nivolumab (anti-CTLA4) antibody) plus ipilimumab (anti-PDL1 antibody) compared to nivolumab alone for patients with rare tumors, including MpBC (NCT02834013)	2	Ongoing
Pembrolizumab (anti-PD1 antibody) plus nab-paclitaxel for TNBC and HR+/HER2− breast cancer cohorts (NCT02752685)	2	Ongoing

## Conclusion

Despite the rarity of MpBC, there are well-established molecular drivers of this aggressive chemoresistant subtype, that is, frequently triple negative. In addition to standard treatments, targeting these molecular alterations, either as monotherapy or in combination, is warranted to improve the dismal prognosis of these patients. Innovative strategies targeting PI3K and NOS as well as immunotherapy and radiation should be tested as rational therapies in patients with MpBC.

## Supplementary information

**Additional file 1.** Supplementary information.

## Data Availability

The dataset generated and analyzed in the current study are available from cBioPortal, https://www.cbioportal.org/ and information on current clinical trials is from https://clinicaltrials.gov/

## Abbreviations

MpBC: Metaplastic breast cancer
TNBC: Triple-negative breast cancer
ER: Estrogen receptor
PR: Progesterone receptor
HER2: Human epidermal growth factor 2
*PIK3CA*: Phosphatidylinositol-4,5-bisphosphate 3-kinase catalytic subunit alpha
TN-IDC: Triple-negative invasive ductal carcinoma
EMT: Epithelial-to-mesenchymal transition
FOXC1: Forkhead Box C1
FOXC2: Forkhead Box C2
Zeb1: Zinc finger E-box-binding homeobox 1
Zeb2: Zinc finger E-box-binding homeobox 2
TIC: Tumor-initiating cell
EGFR: Epidermal growth factor receptor
KIT: Kit proto-oncogene, receptor tyrosine kinase
AKT: Protein kinase B
mTOR: Mammalian target of rapamycin
PIK3R1: Phosphoinositide-3-kinase regulatory subunit 1
TSC2: Tuberous sclerosis complex 2
LKB1: Liver kinase B1
PTEN: Phosphatase and tensin homolog
HR+: Hormone receptor positive
FAT1: Protocadherin fat 1
ARID1A: AT-rich interaction domain 1A
NO: Nitric oxide
iNOS: Inducible nitric oxide synthase
L-NMMA: NG-monomethyl-l-arginine acetate
RPL39: Ribosomal protein L39
STAT3: Signal transducer and activator of transcription 3
Wnt: Wnt family member 1
CTNBB1: Catenin beta 1
APC: Adenomatous polyposis coli
WISP3: Wnt-inducible signaling protein 3
CCN6: Cellular communication of network factor 3
MMTV: Mouse model of mammary epithelium
IGF: Insulin-like growth factor
PD-1: Programmed cell death protein 1
PD-L1: Programmed death ligand 1
TP53: Tumor protein 53
TIL: Tumor-infiltrating lymphocytes
MAPK: Mitogen-activated protein kinase
TERT: Catalytic subunit of telomerase
CDK2NA/p16^INK4a^: Cyclin-dependent kinase inhibitor 2A
CDK2NB/P15^INK4b^: Cyclin-dependent kinase 4 inhibitor B
Cdk: Cyclin-dependent kinase
RT: Radiation therapy
OS: Overall survival
VEGF: Vascular endothelial growth factor
ORR: Overall response rate
CBR: Clinical benefit rate
