# Supplementary material for: A comprehensive overview of metaplastic breast cancer: clinical features and molecular aberrations
Source: Breast Cancer Res. 2020 Nov 4;22:121. doi: 10.1186/s13058-020-01353-z (PMC7640663; doi:10.1186/s13058-020-01353-z)
Supplement: Supplementary file 1 — Additional file 1. Supplementary information. [file 13058_2020_1353_MOESM1_ESM.docx]

**Supplementary Information:**

The ddPCR experiments were performed using a QX200 ddPCR system (Bio-Rad Laboratories, Herculus, California, USA). The ddPCR was run in a final volume of 20 µL with 10 ng PDX tumor DNA, 11 µL ddPCR Supermix for probes (no dUTP), primers for *EGFR* and *RPP30* (reference gene)-targeted amplicons (0.9 µM), 6-carboxyfluorescein (FAM)-labeled probe for *EGFR*-targeted sequence (0.25 µM), and VIC-labeled probe for *RPP30*-targeted sequence (0.25 µM). For droplet generation, 20 µL of sample was added to 70 µL of droplet generation oil. The optimal annealing temperature per application guide of ddPCR was determined using temperature gradient PCR with a row/column of restriction digested 100% wild-type DNA at a relatively high DNA concentration, and concentration gradient PCR was tested using wild-type DNA with mutant spike-in DNA. After droplet preparation, thermal cycling was performed, according to manufacturer’s instructions: 95°C for 10 min, 40 cycles at 94°C for 30 s, 55°C for 1 min, and 98°C for 10 min. The ddPCR CNV analysis was performed using the QuantaSoft software. The EGFR copy number was determined by calculating the ratio of *EGFR* FAM-labeled droplets over the *RPP30* VIC-labeled droplets multiplied by the number of *RPP30* copies (x2 in the human genome).
